# Incidence of Smoking Rates and Relapse During the COVID-19 Pandemic

**DOI:** 10.1192/bjo.2022.249

**Published:** 2022-06-20

**Authors:** Pratyaksha Sinha, Mais Hammoud

**Affiliations:** Weill Cornell Medicine-Qatar, Doha, Qatar

## Abstract

**Aims:**

Along with the numerous structural and cultural changes in healthcare brought about by the COVID-19 pandemic has had an overwhelming psychosocial impact on marginalized communities. Those with substance use disorders (SUD) are a particularly vulnerable group as they are more susceptible to infections and now have less access to healthcare. Smoking cigarettes is known to increase risk of respiratory tract infections due to suppression of respiratory function and impairment of the immune system. It is important to study the smoking rates and relapses over the course of the pandemic to observe whether the increased risk of COVID inflection in smokers and the psychosocial stress of the lockdown have affected the behavior.

**Methods:**

We searched databases including PubMed, PsycArticles, and Cochrane Library using applicable keywords for Substance-Related Disorders, Smoking, and the COVID-19 pandemic lockdown. 69 articles from the results were reviewed, out of which 1 was a meta-analysis and 1 was a systematic review. Other reviews and papers were also consulted to consider case studies and smaller group analyses.

**Results:**

The results of the analysis showed that COVID-19 lockdowns have been negatively affecting those who struggle with substance use disorders, especially that of tobacco use. Evidence suggests a surge of addictive behaviors (both new and relapse) including behavioral addiction over the two years. Recent studies have noted an increase of tobacco use during the pandemic. In some studies, smoking frequency numbers remained stable, and quit attempts decreased.

**Conclusion:**

The psychosocial changes brought on by the pandemic have increased the incidence of smoking frequency and relapse in ex-smokers. This might be due to the increased financial, social, and physical stress, and due to the increased difficulty in accessing healthcare services due to the lockdown. Other stressors contributing to increased smoking could be the stress of contracting a fatal disease, possibility of loss of employment, prolonged confinement, and feeling of boredom. Smoking-related behaviors also increase the risk for contracting the infection due to frequent physical interactions to purchase cigarettes, hand-to-mouth contact when smoking, and increased use of communal smoking places. COVID-19 and addiction are the two pandemics which concurrently can cause a major public health threat. It is important to make the public aware of the increased COVID-19 infection risk attributed to smoking. Hospitals should also resume de-addiction services and provide easy access to advice, support, and pharmacotherapy to those in need.

